# The Impact of Mechanical Debridement Techniques on Titanium Implant Surfaces: A Comparison of Sandblasted, Acid-Etched, and Femtosecond Laser-Treated Surfaces

**DOI:** 10.3390/jfb14100502

**Published:** 2023-10-09

**Authors:** Seung-Mo Eun, Keunbada Son, Sung-Min Hwang, Young-Tak Son, Yong-Gun Kim, Jo-Young Suh, Jun Ho Hwang, Sung-Min Kwon, Jong Hoon Lee, Hyun Deok Kim, Kyu-Bok Lee, Jae-Mok Lee

**Affiliations:** 1 Department of Periodontology, School of Dentistry, Kyungpook National University, Daegu 41940, Republic of Korea; kei963141@gmail.com (S.-M.E.); lhwangl89@naver.com (S.-M.H.); periokyg@knu.ac.kr (Y.-G.K.); jysuh@knu.ac.kr (J.-Y.S.); 2 Advanced Dental Device Development Institute (A3DI), Kyungpook National University, Daegu 41940, Republic of Korea; oceanson@knu.ac.kr (K.S.); dudxkr741@naver.com (Y.-T.S.); 3 Department of Dental Science, Graduate School, Kyungpook National University, Daegu 41940, Republic of Korea; 4 Institute of Advanced Convergence Technology, Kyungpook National University, Daegu 41061, Republic of Korea; hjh@iact.or.kr (J.H.H.); sungmin@iact.or.kr (S.-M.K.); laser@knu.ac.kr (J.H.L.); 5 School of Electronics Engineering, Kyungpook National University, Daegu 41566, Republic of Korea; hdkim@knu.ac.kr; 6 Department of Prosthodontics, School of Dentistry, Kyungpook National University, Daegu 41940, Republic of Korea

**Keywords:** dental implant, femtosecond laser, mechanical debridement, surface roughness

## Abstract

This study evaluated the effects of various mechanical debridement methods on the surface roughness (Ra) of dental implants, comparing femtosecond laser-treated surfaces with conventionally machined and sandblasted with large-grit sand and acid-etched (SLA) implant surfaces. The fabrication of grade 4 titanium (Ti) disks (10 mm in diameter and 1 mm thick) and the SLA process were carried out by a dental implant manufacturer (DENTIS; Daegu, Republic of Korea). Subsequently, disk surfaces were treated with various methods: machined, SLA, and femtosecond laser. Disks of each surface-treated group were post-treated with mechanical debridement methods: Ti curettes, ultrasonic scaler, and Ti brushes. Scanning electron microscopy, Ra, and wettability were evaluated. Statistical analysis was performed using the Kruskal–Wallis H test, with post-hoc analyses conducted using the Bonferroni correction (α = 0.05). In the control group, no significant difference in Ra was observed between the machined and SLA groups. However, femtosecond laser-treated surfaces exhibited higher Ra than SLA surfaces (*p* < 0.05). The application of Ti curette or brushing further accentuated the roughness of the femtosecond laser-treated surfaces, whereas scaling reduced the Ra in SLA surfaces. Femtosecond laser-treated implant surfaces, with their unique roughness and compositional attributes, are promising alternatives in dental implant surface treatments.

## 1. Introduction

Advancements in dental implant technology and scientific studies have culminated in the creation of intricate surface patterns [1]. To promote superior bone–implant contact, aid cell adhesion, and support cell differentiation, rough surfaces are favored over smooth ones [2,3,4]. However, these rough surfaces can inadvertently support bacterial adherence, complicating the effective removal of microorganisms and possibly leading to implant-associated infections and failures [5,6]. Thus, relying exclusively on the mechanical removal of bacteria may not be sufficient [7,8].

Although modern implants offer significant advancements in dental restoration, an alternative to traditional dental prosthetics, ensuring the health of peri-implant tissues remains a hurdle [9]. Plaque control is pivotal for preventing implant-related infections. However, the approaches differ from those for natural teeth because of variations in the surface structures and material compositions of implants [10,11,12]. To promote re-osseointegration, extensive research has explored optimal cleaning methods for contaminated implant surfaces and treating peri-implant bone loss. However, standardized protocols remain elusive [9].

To enhance dental implants biocompatibility, surface treatments enhance material properties while preserving their inherent characteristics. Precise modifications significantly increase surface roughness, achieved through mechanical, chemical, and physical methods [13,14]. For dental implants, such treatments aim to boost wettability by altering surface shape and energy, promote cell proliferation, and expedite osseointegration [15,16,17]. The efficacy of an implant relies on its surface characteristics, and both biocompatibility and surface roughness are paramount for optimal tissue interaction and osseointegration. According to Goyal et al., increased roughness expands the implant’s surface area, facilitating cell migration, attachment, and osseointegration [18,19]. Numerous studies have extolled the virtues of surface treatments on dental implants [15,16,17,18]. Methods such as coating have notably augmented implant surfaces [20]. Titanium and its derived alloys have been identified as the most suitable materials for implant dentistry due to their biocompatibility, corrosion resistance, and the formation of a protective amorphous titanium dioxide (TiO_2_) film on their surface [21]. Titanium (Ti) surfaces treated with plasma spraying present higher surface roughness values than machined surfaces [22]. Moreover, implants with hydroxyapatite (HA) coatings heal faster than their uncoated counterparts [23]. In vitro studies have revealed that acid-etched zirconia implant surfaces have significantly improved cell proliferation, except during initial bone attachment and adhesion phases [24,25,26]. Parsikia et al. achieved superior surface optimization by blasting pure Ti surfaces, followed by a dual-step chemical treatment (acid–alkali) [27], which resulted in enhanced biocompatibility, paving the way for quicker osseointegration. Notably, coarser Ti surfaces catalyzed faster healing than smoother ones [28]. Thus, the role of surface treatment extends beyond preserving implant attributes—it actively fosters and accelerates the healing trajectory.

A surface sandblasted with large-grit sand and acid-etched (SLA) is adopted to induce surface erosion on blasted surfaces [29]. This method combines large-grit sand particles with acid etching to achieve macro-roughness and micro-pits [30], seeking enhanced surface roughness and osseointegration [31,32,33,34]. Cho and Jung [31] identified that SLA surfaces house expansive cavities (diameter of 5–20 µm) and micro-pits (diameter of 0.5–3 µm), leading to augmented surface roughness and area. Consequently, SLA-treated surfaces have been proven beneficial for tissue integration and cell proliferation. The in vivo study on dogs by Xue et al. showed that surfaces subjected to sequential grit blasting and alkaline treatment had superior shear strength, pointing to enhanced early bone growth and osseointegration [35]. Furthermore, research on two-step chemical treatments (acid–alkali) revealed that the finesse in surface morphology, coupled with heightened biocompatibility, facilitates osseointegration during the nascent stages of implantation [36]. According to He et al., implant blasting and subsequent treatment with HCl and H_2_SO_4_ promoted osseointegration during healing, significantly improving biocompatibility [37]. Kim et al. also noted the superior growth of human osteoblast cells on SLA surfaces, attributing this to the increased space available for cell attachment and proliferation [38]. After sandblasting, the SLA surface exhibited a predominantly rough and uneven morphology; however, after acid etching, it attains uniformity punctuated by minuscule micro-pits (diameter 1–2 µm) [38].

Femtosecond laser ablation, prevalent in materials science and life sciences, is pivotal for microfabrication of transparent materials and precision-driven removal of living cells and tissues. Its versatility is also useful in laser tweezer manipulation and multiphoton microscopy. Increased laser output can lead to the irreversible destruction of the target materials. Given its multidisciplinary applicability, research on femtosecond laser ablation is increasing [39]. Most studies on femtosecond lasers concentrate on ascertaining ablation thresholds, evaluating the ablation rate, and using tools such as scanning electron microscopy (SEM) to scrutinize the morphology and temperature dynamics of the ablation area during surgical procedures. In essence, the ablation rate of femtosecond lasers dramatically outpaces picosecond lasers and aligns with mechanical drills. This laser “drill” offers unparalleled precision over traditional drills. SEM and other assays confirm the attainment of crack-free results with resolutions below 10 µm [40]. Additional potential benefits encompass enhanced osseointegration, reduced bacterial adhesion, and increased resistance to biofilm formation [41]. However, experiments related to biofilm formation [42] have shown results similar to SLA surfaces, indicating diverse opinions on the biocompatibility of femtosecond laser-treated surfaces. This suggests the need for further research.

SLA-modified implant surfaces have demonstrated their prowess in stimulating bone cell differentiation and protein synthesis in in vitro and animal studies. Such implants manifest significant bone contact, evidenced by higher torque values upon extraction. Studies have underscored the high success trajectory of SLA-surfaced implants, with success rates soaring past 99% 2 years after a healing duration of approximately 6 weeks [43]. Femtosecond laser-treated implants also exhibit substantial bone–implant contact; however, they are not immune to peri-implantitis. However, comprehensive studies probing the surface alterations post-mechanical treatment on femtosecond laser-treated implant surfaces and juxtaposing them against conventional surfaces are conspicuous by their absence.

Thus, this study aimed to investigate the effects of various mechanical treatments as non-surgical approaches on different implant surfaces commonly used in clinical practice. In clinical settings, the treatment of peri-implantitis often involves the utilization of three primary mechanical debridement tools: the titanium curette, titanium scaler, and titanium brush. Specifically, the study focused on examining their effect on femtosecond laser-treated surface implants and comparing and analyzing their responses with machined and SLA surface implants to assess the influence of surface treatments on different implants. The measurements of the results included the quantification of surface roughness using Ra (average surface roughness) and Sa (average absolute surface roughness), and the assessment of the wetting properties of the specimens through contact angle analysis to examine their hydrophilicity and hydrophobicity. The null hypothesis was that various mechanical treatments did not affect the surface roughness and contact angle of three implant surfaces.

## 2. Materials and Methods

### 2.1. Surface Treatment of Ti Disks

**Figure 1:**
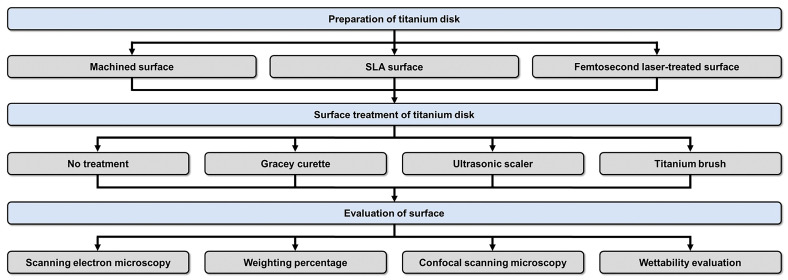
Research flow chart.

The complete sequence of the activities in this study is depicted in Figure 1. The production of Ti disks and the SLA process were conducted by a dental implant manufacturer (DENTIS; Daegu, Republic of Korea). The Ti disks (grade 4) consisted of 0.08% carbon (C), 0.5% iron (Fe), 0.015% hydrogen (H), 0.05% nitrogen (N), 0.40% oxygen (O), and 98.9% titanium (Ti). To achieve a smooth surface with minimal roughness, grade 4 Ti disks (10 mm in diameter, 1 mm in thickness) underwent a machining procedure using a CNC milling machine. The selection of cutting parameters and tools aimed to reduce surface defects and achieve the desired surface quality. These Ti disks, manufactured using this method, were categorized as the machined surface group. Due to confidentiality concerns, obtaining detailed information about the technology from the manufacturer proved challenging. Ti disks were exposed to large-grit alumina particles (250–500 µm) through a sandblasting machine at a pressure of 4 bars for 20 s. Subsequently, the disks underwent acid etching in a mixture of HCl and H_2_SO_4_ (1:1 *v*/*v*) for 30 min at 60 °C. They were then rinsed with distilled water and dried at room temperature. Ti disks manufactured using this process were classified as the SLA surface group. In this study, the modification of milled Ti disk surfaces to exhibit hydrophilic properties was investigated using an ultrashort pulse multiwavelength laser (femtosecond laser). Specifically, a laser with a 343-nm wavelength was applied with a scanning speed of 10 mm/s and a repetition rate of 200 kHz to create a line pattern with a 50-µm pitch distance on both Ti and ceramic specimens. The resulting Ti disks fabricated were categorized as the femtosecond laser-treated group.

To investigate the effects of treatments on various implant fixture surfaces, three types of fixture disks were prepared: machined (6 disks), SLA (6 disks), and femtosecond laser-treated (6 disks) implant surfaces. For each implant type, saline was irrigated using a curette at a rate of 1 stroke per second for 10 strokes. All other treatments were performed for 10 s [9]. The treatment was performed by a single experimenter to simulate an environment similar to actual clinical conditions. Prior to this, the experimenter underwent sufficient practice to ensure the application of a force comparable to that used in real clinical settings.

**Figure 2:**
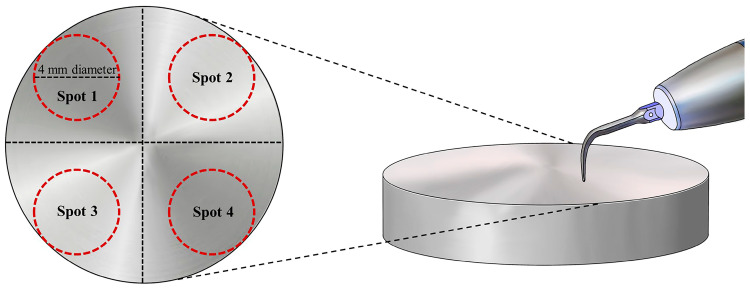
Schematic of the disk surfaces where post-treatments were performed. Spot 1, control group; spot 2, test group 1; spot 3, test group 2; spot 4, test group 3.

**Figure 3:**
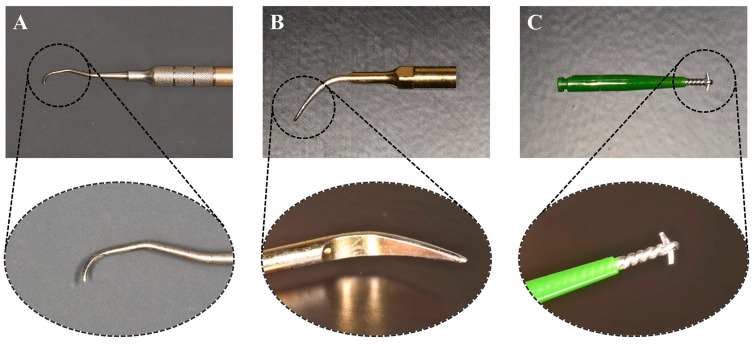
Medical devices used for mechanical debridement treatment. (**A**) Gracey curette. (**B**) Ultrasonic Ti scaler. (**C**) Titanium brush.

The control group did not receive treatment (Figure 2). In test group 1 (Figure 2), the Ti implant surface was treated with a Gracey curette (Atria, Seoul, Republic of Korea) (Figure 3). For test group 2 (Figure 2), an ultrasonic scaler (Woodpecker, Guilin, China) was used, and test group 3 was treated using a Ti brush (Neobiotech, Seoul, Republic of Korea) (Figure 3).

### 2.2. SEM, Weight Percentage, and Confocal Scanning Microscopy

The treated surfaces were analyzed using an SEM (S-4800, Hitachi, Ltd., Tokyo, Japan) at magnifications between 10× and 100× to evaluate surface roughness and compositional changes. Surface damage, as revealed by SEM, was classified as (1) minimal or no damage, (2) moderate damage, and (3) severe damage in relation to the control group. Two dentists independently assessed the surface damage. Discrepancies were resolved through discussion until consensus was achieved [9].

For compositional change evaluation, energy-dispersive spectrometry (EDS) analyzed the chemical characteristics of the specified implant regions. Atomic and weight percentages of carbon, oxygen, and Ti were determined using a field-emission SEM (S-4800, Hitachi, Ltd., Tokyo, Japan) [9].

Surface roughness was gauged by measuring the Ra and Sa values for each specimen using a confocal laser scanning microscope (OLS-4100, Olympus, Japan) at 10× magnification [44,45]. The extent of surface damage was statistically compared between the control and experimental groups based on the acquired Ra and Sa values [9].

### 2.3. Wettability Evaluation

To determine the interfacial properties post-mechanical treatments, contact angles were measured using a contact angle analyzer (Phoenix-MT; SEO, Suwon, Republic of Korea). Distilled water droplets were placed on treated specimens, and the angles formed between the specimen and the water droplet were measured five times from both sides. The mean contact angle was subsequently calculated.

### 2.4. Statistical Analysis

Statistical analysis was executed using IBM SPSS Statistics version 26 (IBM Corp., Armonk, NY, USA). Given the data’s nature, nonparametric tests were chosen for analysis. To discern significant differences in roughness and contact angle across the three implant surface treatments, the Kruskal–Wallis H test was utilized (α = 0.05). If significant variances were noted, pairwise comparisons were made with Bonferroni correction to manage multiple comparisons. Differences among implant surface treatments were highlighted with uppercase letters (α = 0.05).

## 3. Results

### 3.1. SEM Analysis

**Figure 4:**
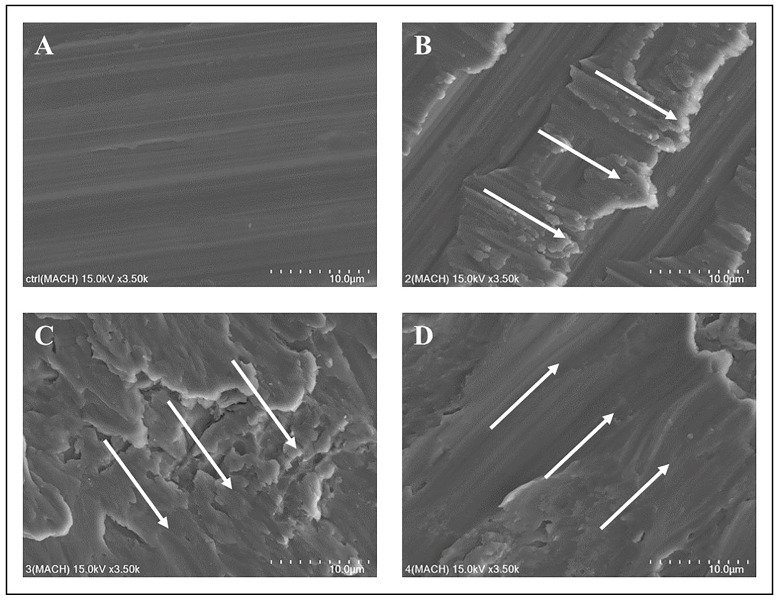
SEM images of the machined surface: (**A**) SEM image of the machined surface, control group; (**B**) machined surface treated with a titanium curette; (**C**) machined surface treated with a titanium scaler; (**D**) machined surface treated with a titanium brush. The white arrow indicates the direction in which the mechanical debridement treatment was performed. Each scale bar represents 10 µm.

SEM was utilized to discern damage patterns on the implant surfaces post-mechanical treatments. The machined surface (control) group displayed an undisturbed surface (Figure 4A). The Ti curette-treated machined surface exhibited moderate damage in areas not contacted by the curette (Figure 4B). The machined surface displayed enhanced roughness after this treatment. The Ti scaler-treated group exhibited extensive surface deformation, obfuscating the original machined surface (Figure 4C). This deformation was substantial, presenting an irregular surface topography. Conversely, the Ti brush-treated group displayed uniform directional patterns, which were attributed to the rotational motion of the Ti brush, with some surface deformities (Figure 4D).

**Figure 5:**
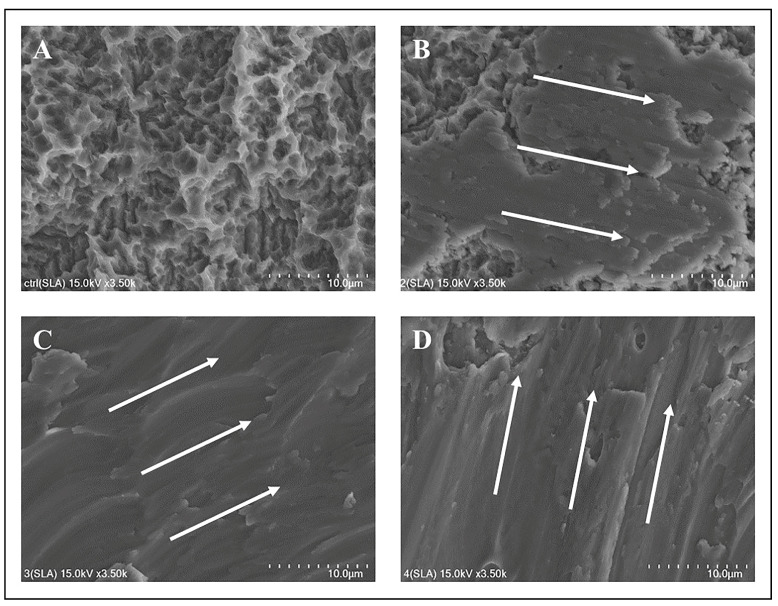
SEM images of the SLA surface: (**A**) SEM image of the SLA surface, control group; (**B**) SLA surface treated with a titanium curette; (**C**) SLA surface treated with a titanium scaler; (**D**) SLA surface treated with titanium brush. The white arrow indicates the direction in which the mechanical debridement treatment was performed. Each scale bar represents 10 µm.

The SLA surface (control) group demonstrated macro-roughness from large-grit sandblasting, complemented by micro-roughness from acid etching (Figure 5A). The Ti curette-treated SLA group displayed diminished roughness (Figure 5B). Notably, the Ti scaler induced significant deformation on the SLA surface, which decreased its inherent micro-porosity. Similar surface deformations with the Ti brush were observed for both machined and SLA surfaces, signifying uniformity in results.

**Figure 6:**
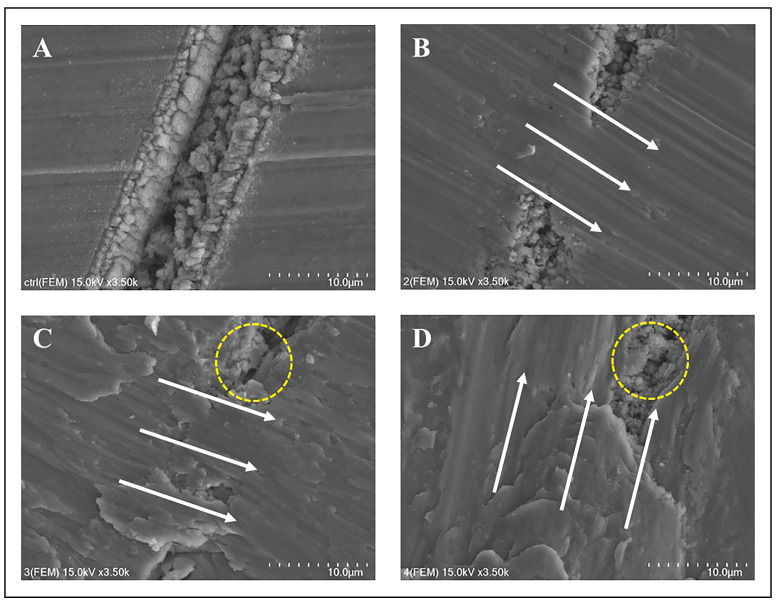
SEM images of the femtosecond laser-treated surface: (**A**) SEM image of the femtosecond laser-treated surface, control group; (**B**) femtosecond laser-treated surface treated with a titanium curette; (**C**) femtosecond laser-treated surface with a titanium scaler; (**D**) femtosecond laser-treated surface with a titanium brush. The white arrow indicates the direction in which the mechanical debridement treatment was performed. Each scale bar represents 10 µm.

The femtosecond laser-treated (control) group showed heightened roughness (Figure 6A). Following the treatment with the Ti curette (Figure 6B), surface deformation patterns were congruent with those observed on machined and SLA surfaces. Treatment with the Ti scaler (Figure 6C) and Ti brush (Figure 6D) yielded similar outcomes.

### 3.2. Elemental Weight Percentages

Table 1 presents the weight percentages of carbon, oxygen, Ti, and aluminum for each specimen pre- and post-mechanical treatments. Irrespective of surface or treatment type, composition ratios remained relatively consistent across specimens.

### 3.3. Confocal Laser Scanning Microscopy

**Figure 7:**
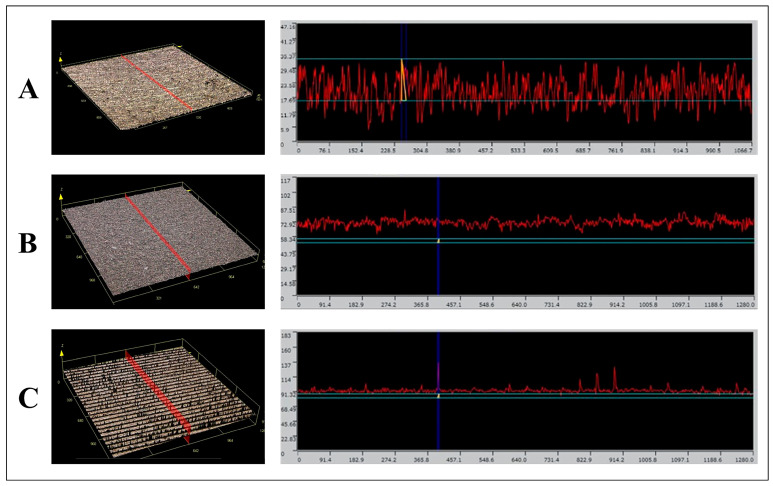
Representative image of surface morphology of the control group using confocal laser scanning microscopy. (**A**) Machined group. (**B**) SLA group. (**C**) Femtosecond laser-treated group.

The surface roughness and morphology of the samples were evaluated by confocal laser scanning microscopy (Figure 7). A significant variance in surface roughness values (Ra and Sa) was observed based on the mechanical treatments applied. The Ti brush-treated group consistently recorded the highest roughness values. No significant roughness difference was observed among the mechanical treatments for the femtosecond laser-treated and machined groups. Conversely, the SLA group, when treated with a Ti scaler, exhibited a markedly reduced Ra value.

### 3.4. Surface Roughness

The statistical significance of results, based on different surface treatment methodologies and types of surfaces, was evaluated using the Kruskal–Wallis H test. For post-hoc comparisons among groups, either by surface treatment methods or by surface types, the Bonferroni correction method was utilized (Table 2 and Table 3).

In the control group, the Ra value (a measure of surface roughness) showed no significant difference between the machined surface group and the SLA group (*p* > 0.05). Conversely, the surface roughness of the femtosecond laser-treated group was greater than that of the SLA group (*p* < 0.05; Table 2).

Upon mechanical treatment using a Ti curette, the femtosecond laser-treated group exhibited a markedly increased Ra value in comparison with other samples. Meanwhile, the machined and SLA surfaces did not display any significant difference in roughness (Table 2).

When subjected to treatment with a scaler, the SLA group manifested a notable reduction in the Ra value, whereas the femtosecond laser-treated group showed a significant increment (Table 2). Lastly, when treated with a Ti brush, the femtosecond laser-treated group displayed a higher Ra value than the SLA group (Table 2).

### 3.5. Contact Angle

**Figure 8:**
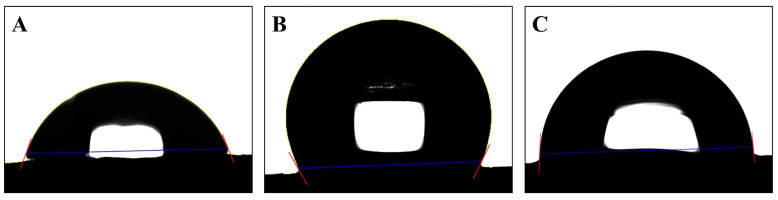
Representative image of surface contact angle of the control group. (**A**) Machined group. (**B**) SLA group. (**C**) Femtosecond laser-treated group.

Table 4 presents comparisons of surface contact angles across different implant surface treatments (Figure 8). The machined group, when treated with the Ti brush, exhibited a significantly reduced contact angle compared with other treatments. The contact angle of the SLA group significantly varied based on the treatment applied.

## 4. Discussion

### 4.1. SEM Analysis

For the machined group, the Ti scaler induced pronounced surface deformations, rendering the surface nearly unrecognizable. This extensive deformation is attributable to the increased frequency and intensity of scaler–sample contact compared with the Ti curette. The intense vibrations of Ti scalers compromised the original structure of the machined surface.

The SLA group displayed greater surface alteration with the Ti scaler than with the Ti curette. Notably, the deformed SLA surface was comparable to the machined surface. The nature and directionality of these deformations likely resulted from the technique employed by the experimenter using the Ti scaler.

Observations of the femtosecond laser-treated surface mirrored these findings. SEM images showed no significant variance in the patterns and characteristics of surface alterations among sample types. Thus, the response of the femtosecond laser-treated group to mechanical treatment paralleled the other two groups.

Consistent with previous studies, different mechanical treatment modalities induced significant surface transformations, particularly when using an ultrasonic Ti scaler and Ti brush. When compared to results from other studies, similar surface transformation patterns were observed [44]. In this study, when using a Ti scaler and Ti brush, results indicated an irregular pattern on the surface, and similarly, in this experiment, similar changes were observed when a Ti scaler was used. When a Ti brush was used, the alteration in surface pattern changes was also comparable to this experiment. This underscores the point mentioned in the referenced paper that mechanical treatment can potentially induce damage to the implant surface. Notably, similar findings emerged in the femtosecond laser-treated group. The Ti curette produced predictable patterns of surface alterations, which can be attributed to the operator’s technique. By contrast, the ultrasonic Ti scaler, with its intricate motions, yielded diverse damage patterns, leading to inconsistent surface deformities. As the Ti brush operated with a steady rotational direction, uniform alterations in surface patterns were anticipated.

Comparing all surfaces revealed that independent of the surface type, mechanical treatment yielded analogous surface alteration patterns. With previous research, this alignment extends to the mechanical treatment outcomes of the femtosecond laser-treated group.

### 4.2. Weight Percentage of Carbon, Oxygen, Ti, and Aluminum Atoms

Compositionally, the machined, SLA, and femtosecond laser-treated groups showed negligible differences. Given that SLA specimens are derived from machined group specimens and femtosecond laser treatment does not substantially alter the composition, this consistency is expected. However, the femtosecond laser-treated group exhibited a slightly high oxygen proportion, likely due to Ti oxidation during laser treatment.

Aluminum was noted in specimens treated with the Ti curette, attributable to aluminum content in the curette itself. Thus, compositionally, no remarkable disparities were found when comparing machined and SLA surfaces.

According to Kim et al. [45], Ti primarily exists as TiO_2_, a highly reactive form [46]. EDS analysis signals confirm that the Ti disk surface comprises a Ti oxide layer [13]. This oxide layer typically ranges from 2 to 6 nm, whereas EDS analysis furnishes compositional data up to a depth of 1 mm. After the SLA surface undergoes acid etching to eliminate residual Ti surface elements, as indicated in Table 1, the femtosecond laser-treated Ti surface reacts more vigorously with oxygen than its counterparts. Consequently, discernible differences in Ti, oxygen, and carbon elemental ratios emerge between studied groups, persisting post-mechanical treatment. It is plausible to infer that femtosecond laser treatment induces chemical compositional shifts on the surface of the Ti disk.

### 4.3. Confocal Laser Scanning Microscopy and Surface Roughness

Mechanical interventions using the Ti curette, Ti scaler, and Ti brush consistently induced surface damage across all surfaces, including the machined, SLA, and femtosecond laser-treated surfaces. In particular, the Ti brush significantly compromised the implant fixture’s surface integrity. From a statistical standpoint concerning surface roughness, only the Ti scaler’s application to the SLA surface notably decreased the Ra value. By contrast, other mechanical interventions did not considerably affect surface roughness.

In intra-group comparisons across machined, SLA, and femtosecond laser-treated groups, minimal differences emerged, with the notable exception of the SLA group subjected to the Ti scaler, which registered a significantly diminished Ra value. With pre-mechanical treatment, the surface roughness of the femtosecond laser-treated group did not vary substantially; however, post-treatment comparisons with other groups revealed marked differences.

In summary, while mechanical treatments undeniably induced surface alterations, only the SLA surface subjected to the Ti scaler registered a decline in existing surface roughness values.

### 4.4. Contact Angle

Studies assessing contact angles—indicative of specimen hydrophilicity—revealed that the femtosecond laser-treated group surpassed the SLA surface in hydrophilicity. A sub-90-degree water droplet contact angle denotes material hydrophilicity [47]. Thus, while the SLA surface displayed hydrophobic traits, the femtosecond laser-treated surface leaned toward hydrophilicity. This enhanced hydrophilicity might foster superior biological environment interactions [48], expediting osseointegration, a pivotal factor [49]. Thus, it is conceivable that femtosecond laser treatments might amplify implant surface hydrophilicity, promoting quicker bone fusion. Hydrophilicity presents major advantages during the initial stages of wound healing and during the cascade of events that occur during osseointegration, facilitating bone integration. In this regard, this represents a potential biological benefit [48].

Recent literature highlights that laser-modified implant surfaces offer optimal surface texture with minimal contamination relative to other techniques. A distinct advantage of laser technology lies in its applicability for texturing implants with intricate designs. Femtosecond lasers have an edge over nanosecond lasers, boasting superior precision, minimized heat effect zones, and fewer residual particles. This technological advance holds the potential for substantially streamlining conventional surface treatments.

Initial endeavors to texture Ti using lasers employed long-pulse lasers. Such laser-modified implant surfaces present the dual benefits of ideal surface micro-roughness and minimal contamination. Femtosecond laser treatments can craft a spectrum of micro-roughness typologies, primarily bifurcated into two categories. The first type features micro-roughness spanning from 1 to 15 µm, with characteristics, dimensions, and compositions modulated by laser fluence and shot counts. This micro-roughness type appears unique to femtosecond laser treatments. The second category entails smoother surfaces punctuated by minor irregularities, manifesting at peak laser fluence levels when melting engulfs the entire targeted zone. Quick solidification of these molten surfaces results in smooth, microscale roughness, even following maximal laser fluence treatment.

Femtosecond laser treatments not only produce nanostructures but also craft a broad array of microscale structures alongside a blend of micro- and nanostructures [50]. Accordingly, femtosecond laser-treated Ti implants might supersede traditional implants, provided their mechanical treatment outcomes align with extant machined and SLA surfaces. However, a deeper dive into biological studies is needed to explore biological responses to unique microstructures inherent to femtosecond laser-treated implants. This includes probing bone–implant contact dynamics, interactions with living cells, bacterial adherence alterations due to varying surface energies from different treatments, oral bacterial attachment shifts, and bacterial strain modifications. Additionally, it is worth considering that conducting experiments involving additional diverse mechanical treatment methods (e.g., plastic curette, Teflon tip, air abrasives, etc.) could provide valuable insights for referencing outcomes from various mechanical treatments in clinical settings. Furthermore, there is a lack of research on the subsequent biological responses when mechanical treatment is applied to implants with conventional surfaces compared to implants treated with femtosecond laser surface modifications. Conducting further studies in this regard is deemed desirable.

## 5. Conclusions

Femtosecond laser-treated implant surfaces exhibited results comparable to those of conventionally treated implant surfaces. Of the various treatment methods assessed, only the Ti scaler demonstrated a significant reduction in surface roughness, whereas other treatments resulted in minimal changes to roughness levels. While compositional differences exist between the femtosecond laser-treated group and the traditional machined and SLA groups, the degree of these differences was not markedly disparate across treatments. Most notably, the femtosecond laser-treated group displayed enhanced hydrophilicity, as evidenced by its contact angle, relative to the SLA group. Given the potential biological benefits of femtosecond laser-treated implants, they might emerge as viable alternatives to traditional implant surface treatments in the future.

## Figures and Tables

**Table 1:** Weight percentage of carbon, oxygen, titanium, and aluminum atoms.

Group	Method	Weight Percentage			
		C	O	Ti	Al
Machined group	Control	1.2	5.05	93.75	-
	Titanium curette	1.06	6.26	91.93	0.74
	Titanium scaler	0.94	9.03	90.02	-
	Titanium brush	1.01	5.44	93.55	-
SLA group	Control	0.55	4.83	94.61	-
	Titanium curette	1.76	7.18	87.88	3.19
	Titanium scaler	0.6	5.19	94.2	-
	Titanium brush	1.04	4.71	94.24	-
Femtosecond laser-treated group	Control	1.64	19.32	79.05	-
	Titanium curette	1.21	13.07	85.52	0.2
	Titanium scaler	1.25	11.06	87.68	-
	Titanium brush	1.26	18.34	80.41	-

**Table 2:** Comparison of roughness (µm, Ra) on the different implant surface treatments.

Roughness Type	Surface Treatment	Surface Type	Mean	SD	95% Confidence Interval		*p* *	Comparison **
					Lower	Upper		
Ra	Control	Machined	3.374	1.662	1.630	5.118	<0.05	AB
		SLA	2.536	0.240	2.284	2.788		A
		Femto	5.610	2.765	2.708	8.511		B
	Titanium Curette	Machined	3.124	1.893	1.137	5.111		A
		SLA	2.796	0.210	2.576	3.016		A
		Femto	5.786	2.156	3.524	8.048		B
	Titanium Scaler	Machined	3.911	1.721	2.105	5.717		A
		SLA	2.143	0.288	1.840	2.445		B
		Femto	5.822	0.790	4.993	6.650		C
	Titanium Brush	Machined	4.243	1.751	2.405	6.081		AB
		SLA	2.694	1.046	1.596	3.791		A
		Femto	6.103	0.735	5.332	6.874		B

* Significant difference in the roughness of the different surface treatments determined at different surface types using the Kruskal–Wallis H test, *p* < 0.05. ** Significant differences among different implant surface treatments are indicated by different capital letters using the Bonferroni correction, *p* < 0.05.

**Table 3:** Comparison of roughness (µm, Sa) on different implant surface treatments.

Rough-ness Type	Surface Treatment	Surface Type	Mean	SD	95% Confidence Interval		*p* *	Comparison **
					Lower	Upper		
Sa	Control	Machined	0.204	0.109	0.090	0.318	<0.001	A
		SLA	1.698	1.127	0.516	2.881		A
		Femto	5.380	2.570	2.683	8.077		B
	Titanium Curette	Machined	0.255	0.142	0.106	0.404		A
		SLA	1.876	1.246	0.568	3.184		A
		Femto	5.553	2.153	3.293	7.813		B
	Titanium Scaler	Machined	0.363	0.205	0.148	0.577		A
		SLA	1.234	0.856	0.336	2.132		A
		Femto	6.083	0.538	5.518	6.648		B
	Titanium Brush	Machined	0.378	0.199	0.170	0.586		A
		SLA	2.151	1.167	0.927	3.375		B
		Femto	6.214	0.575	5.611	6.817		C

* Significant difference in the roughness of different surface treatments determined at different surface types using the Kruskal–Wallis H test, *p* < 0.05. ** Significant differences among different implant surface treatments are indicated by different capital letters using the Bonferroni correction, *p* < 0.05.

**Table 4:** Comparison of the surface contact angles on different implant surface treatments.

Surface Type	Treatment Type	Mean	SD	95% Confidential Interval		*p* *	Comparison **
				Lower	Upper		
Machined	Control	72.837	5.302	69.901	75.773	0.012	A
	Curette	73.294	2.563	71.875	74.713		A
	Scaler	73.061	5.429	70.054	76.067		A
	Brush	67.712	6.679	64.013	71.411		B
SLA	Control	99.883	12.482	92.971	106.795	<0.001	A
	Curette	80.739	11.713	74.252	87.225		B
	Scaler	75.317	14.249	67.427	83.208		B
	Brush	73.865	9.564	68.568	79.161		B
Femtosecond laser	Control	94.073	5.926	90.792	97.355	<0.001	A
	Curette	96.100	7.931	91.708	100.492		A
	Scaler	86.287	3.826	84.169	88.406		B
	Brush	85.253	5.593	82.156	88.350		B

* Significant difference in the surface contact angle of different surface treatments determined at different surface types using the Kruskal–Wallis H test, *p* < 0.05. ** Significant differences among different implant surface treatments are indicated by different capital letters using the Bonferroni correction, *p* < 0.05.

## Data Availability

The datasets used and/or analyzed during the current study are available from the corresponding author on reasonable request.
